# Risk prediction models for cognitive impairment in patients with chronic kidney disease: a systematic review

**DOI:** 10.3389/fpubh.2026.1851336

**Published:** 2026-06-11

**Authors:** Lei Dong, Yun Chen, Xiaohong Lin, Jiyuan Shi, Hongxia Liu, Yinqin Zhong

**Affiliations:** 1 School of Nursing, Beijing University of Chinese Medicine, Beijing, China; 2 Shenzhen Hospital (Futian) of Guangzhou University of Chinese Medicine, Shenzhen, China

**Keywords:** chronic kidney disease, cognitive impairment, dialysis, risk prediction model, systematic review

## Abstract

**Introduction:**

Prediction models for cognitive impairment offer significant advantages in public health preventive strategies in high-risk chronic kidney disease populations, but their methodological quality and applicability remain unclear. The aims of this study were to systematically review and critically appraise the existing prediction models for cognitive impairment in chronic kidney disease.

**Methods:**

PubMed, Web of Science, Embase, CINAHL, Cochrane Library, China National Knowledge Infrastructure, Wanfang Database, SinoMed, and China Science and Technology Journal Database were searched from inception to 12 October 2025. The prediction model risk of bias assessment tool was used for quality assessment, and the checklist for critical appraisal and data extraction for systematic reviews of prediction modeling studies was used for data extraction. A narrative synthesis was performed to summarize the characteristics, performance, and risk of bias of existing models. Subgroup analyses were performed stratified by outcome definitions, CKD population, and cognitive assessment tools.

**Results:**

A total of 5,972 studies were identified, ultimately including 17 models for three outcomes: cognitive impairment (52.9%), mild cognitive impairment (11.8%), and cognitive frailty (35.3%). The reported prevalence ranged from 12.8 to 77.9% for cognitive impairment, around 50% for mild cognitive impairment, and 14.2–25.8% for cognitive frailty. Most models (58.8%) were developed in hemodialysis populations. Fifteen studies were internally validated, and 7 were externally validated. For internal validation, the AUC of cognitive impairment models ranged from 0.745 to 0.918, mild cognitive impairment models from 0.926 to 0.928, and cognitive frailty models from 0.842 to 0.945. For external validation, the AUC of cognitive impairment models ranged from 0.752 to 0.899, the mild cognitive impairment model was 0.897, and cognitive frailty models ranged from 0.832 to 0.904. All studies were rated as high risk of bias, mainly due to methodological problems in the analysis domain, with great concerns regarding applicability in 13 studies.

**Conclusion:**

Current research on prediction models for cognitive impairment in chronic kidney disease remains in the developing stage, with heterogeneous outcome definitions and all studies at high risk of bias. Most studies are limited by methodological shortcomings.

**Systematic review registration:**

https://www.crd.york.ac.uk/PROSPERO/view/CRD420251007577, Identifier CRD420251007577.

## 1 Introduction

Cognitive impairment (CI) associated with chronic kidney disease (CKD) represents a growing public health burden linked to increased medical costs, adverse health outcomes, and reduced quality of life. CI presents as an insidious and progressive cognitive decline, characterized by memory impairment, attention deficits, communication difficulties, and executive dysfunction (1). With CKD progression, CI becomes increasingly prevalent, with reported rates ranging from 47.3 to 60.6% (2). It worsens adherence to medical treatment and exacerbates adverse health outcomes, especially for CKD patients who necessitate long-term volume management, dietary restrictions, medication adherence, and routine clinical monitoring (1). Besides, CI was proven to be significantly associated with frailty, anxiety, depression, sleep disorders, and reduced quality of life, which imposes an increased medical burden and healthcare resource utilization (3, 4). Beyond a certain threshold of progression, CI often becomes irreversible. However, early manifestations of CI are subtle and insidious, leading to diagnostic and therapeutic delays (5). Accordingly, it is critical to identify and adopt effective measures to prevent CI among CKD patients.

Early, individualized, and accurate risk prediction is a critical step in CI prevention. Therapeutic interventions yield superior outcomes earlier than more advanced stages of CI in CKD. Prediction models can integrate multiple predictors to estimate the probabilities of specific outcomes (6). It enables healthcare professionals to identify individuals at high risk and initiate appropriate interventions based on stratified risk levels (7). Despite the development of some risk prediction models for CI in patients with CKD (8–10), evidence on their methodological quality, predictive performance, and applicability in clinical practice remains limited and inconclusive. No consensus has been reached regarding the optimal prediction model for CI risk in individuals with CKD. In addition, different predictors such as demographic, psychosocial, and clinical variables, as well as indicators from laboratory tests, affect CI (8, 11, 12), and it is essential to characterize these factors for better prevention of CI in high-risk CKD populations.

A systematic evaluation of the prediction model performance is essential to examine its predictive ability across diverse study settings, populations, or locations, and to assess the need for further model refinement or improvement (13). The demand for evidence synthesis in the (external validation) research on the performance of the model in new individuals is increasing. However, there is a lack of quantitative synthesis or meta-analysis of specific models’ prediction performance, with potential reasons for this gap including a lack of methodological guidance, unavailability of relevant summary statistics, or concerns about the quality of included studies (13).

This study aims to systematically review the characteristics and performance of existing prediction models for CI risk in patients with CKD, to critically appraise the risk of bias (ROB) and clinical applicability. Additionally, we will summarize the major predictive factors for CI among CKD patients to provide valuable references for clinical practice.

## 2 Methods

### 2.1 Study design

This systematic review followed the transparent reporting of multivariable prediction models for individual prognosis or diagnosis in systematic reviews and meta-analyses (14). The checklist for critical appraisal and data extraction for systematic reviews of prediction modeling studies (15) was used to extract data. The study has been registered with PROSPERO (registration No: CRD420251007577).

The PICOTS framework recommended by the guidelines was systematically applied to define the research question, to develop the comprehensive search strategy, and to establish explicit inclusion and exclusion criteria for study selection (13, 15). The key elements are described as follows: P (Population): CKD patients aged 18 or older, defined as a low GFR (<60 mL/min/1.73 m^2^) or kidney damage present for more than 3 months (16); I (Intervention model): risk prediction model development with or without external validation in independent data for CI in patients with CKD (predictors ≥ 2); Comparator (C): no competing model; Outcome (O): CI, regardless of the degree; Timing (T): CI was assessed during hospitalization or outpatient visits, covering all disease stages without imposing time constraints; Setting (S): the intended use of the prediction model is to stratify the risk of CI in patients with CKD, thereby supporting the formulation of preventive strategies to reduce adverse outcomes.

### 2.2 Search strategy

A comprehensive search was conducted on PubMed, Web of Science, Embase, CINAHL, Cochrane Library, China National Knowledge Infrastructure, Wanfang Database, SinoMed, and China Science and Technology Journal Database to identify relevant studies published from the inception of the databases to October 12th, 2025, with language restriction to English and Chinese. The search terms included “chronic kidney disease,” “renal insufficiency, chronic,” “chronic kidney insufficiency,” “chronic renal disorder,” “renal failure,” “end stage renal disease,” “dialysis,” “progressive kidney,” “prediction model,” “risk factor,” “predictor,” “risk score,” “risk assessment,” “nomogram,” “cognition,” “cognitive dysfunction,” “cognitive impairment,” “cognitive disorder,” “mental deterioration” (Supplementary Table 1). The reference lists of included studies were manually searched for potentially relevant studies.

### 2.3 Inclusion and exclusion criteria

The studies were included with: (1) involving CKD patients aged 18 or older; (2) observational study designs including prospective cohort, cross-sectional, and case–control designs; (3) risk prediction model development with or without external validation in independent data; and (4) the outcome of CI, regardless of the degree. The following exclusion crite ria were considered: (1) studies evaluating fewer than 2 predictor variables for CI; (2) studies with unavailable full text and information; (3) no model performance evaluation, such as area under the receiver operating characteristic curve (AUC), C-statistic, calibration plot, calibration slope, Hosmer–Lemeshow test, Brier score, and *R*^2^; (4) conference abstracts or letters to the editor; and (5) duplicate publications.

### 2.4 Study selection

Firstly, the retrieved records were imported into EndNote software, and duplicates were removed. Then, two independent reviewers independently excluded irrelevant studies by reading the titles and abstracts, based on predefined inclusion and exclusion criteria. Finally, the full text of the remaining reviews was determined to be the final eligibility criterion. Any disagreements were resolved through consensus between the reviewers or consultation with the third reviewer.

### 2.5 Data extraction

The main reviewer extracted data from the included studies, and then the second reviewer double-checked to ensure accuracy. The following information were included according to the checklist for critical appraisal and data extraction for systematic reviews of prediction modeling studies: (1) Basic information: author, publication year, country, participants, study design, data source, main outcome, CI cases, and sample size; (2) Model information: handling of missing data, final predictors in the model, model development (including modeling method and method for selection of predictors during multivariable modeling), model validation (internal and/or external validation), model performance (discrimination and calibration), other indexes, and formats of model presentation. The desired but unreported data were obtained by calculating from other reported information or contacting authors.

### 2.6 Quality assessment

Two reviewers independently assessed the ROB and applicability of the included models using the Prediction Model Risk of Bias Assessment Tool (PROBAST) (17). PROBAST is used to assess the development, validation, or updating of multivariate predictive models for prognosis or diagnosis. This tool evaluates the ROB in four key domains: participants, predictors, outcome, and analysis, with 20 specific signaling questions. Each signaling question is answered with “yes (Y),” “probably yes (PY),” “no (N),” “probably no (PN),” or “no information (NI).” A domain where an answer of N or PN on 1 or more signaling questions should be judged as high ROB. The overall ROB is considered low risk if all four domains are at low risk. The applicability was evaluated for the first three domains using an approach analogous to ROB assessment, with no signaling questions assigned to each domain. The degree of overall ROB and applicability is rated as “low,” “high,” or “unclear” concern.

### 2.7 Data synthesis

Analyses were implemented using the Stata software. We performed a narrative analysis of the enrolled studies to synthesize key information (characteristics, model performance, and ROB) of the predictive models. Model discrimination was primarily evaluated by AUC or C-statistic. Calibration was assessed using the Hosmer–Lemeshow test, calibration plots, or Brier score. Some studies could report the decision curve analysis (DCA) of the predictive models. To address the issues of clinical and methodological heterogeneity, we conducted three pre-specified subgroup analyses (outcome definitions, CKD population, and cognitive assessment tools) to stratify model performance or predictor frequency. No quantitative analysis of the results was performed.

## 3 Results

### 3.1 Study selection

**Figure 1:**
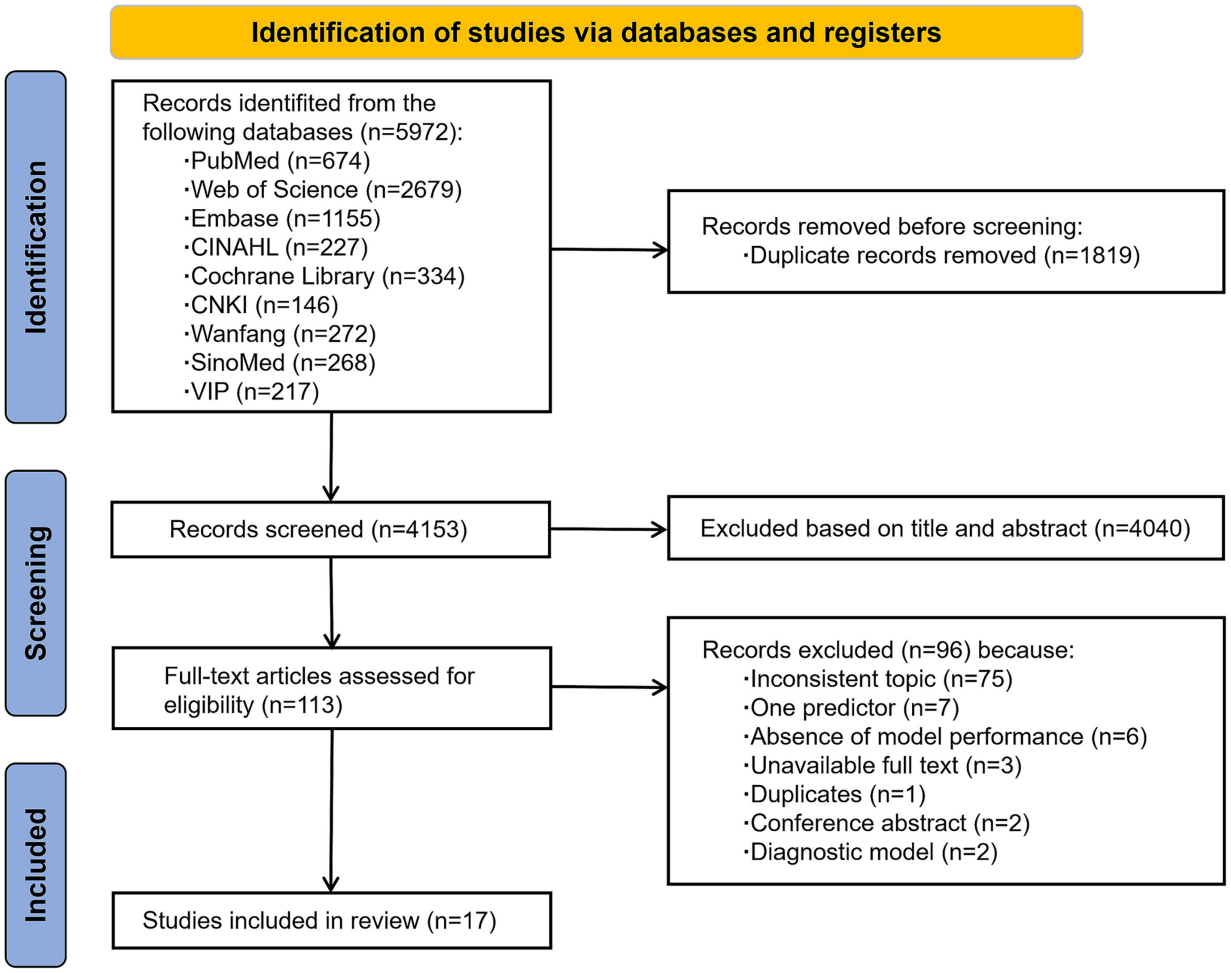
Preferred Reporting Items for Systematic reviews and Meta-Analyses (PRISMA) flowchart of studies search and selection.

The detailed process of study search and selection is shown in Figure 1. A total of 5,972 indexed records were retrieved through the initial search. A total of 17 studies (8–12, 18–29) with 17 models were included in the final systematic review. Among them, one study identified the optimal ensemble model based on five individual models using machine learning algorithms- support vector machine, extreme gradient boosting, random forest, neural network, and logistic regression (8). Four machine learning algorithms were used to develop a CI risk prediction model in another study (28).

### 3.2 Study characteristics

Table 1 summarizes the designs and participant characteristics of the 17 included studies. The studies were published between 2022 and 2025, among which 16 were conducted in China, and one (22) from the NHANES database. The sample sizes of the included studies ranged from 50 to 1,075. Regarding the study subjects, 11 studies focused on end-stage renal disease (ESRD), such as hemodialysis (8, 12, 18, 20, 21, 23, 24, 26, 27, 29), peritoneal dialysis (25). Approximately a quarter of the prediction models (*n* = 4, 23.5%) only focused on older patients (8, 9, 19, 22).

**Table 1:** Basic characteristics of included studies.

Author (year)	Country	Study design	Participants	Data source	Main outcome	CI cases/sample size (%)
Cao (2025) (8)	China	Cross-sectional survey	Patients with maintenance hemodialysis treated for more than 3 months, ≥60 years old	Several hospitals	Cognitive frailty (MoCA and FP scale)	153/1075 (14.2%)
Cao (2025) (28)	China	Retrospective study	Patients with chronic kidney disease	The China health and retirement longitudinal study	Cognitive impairment (Health status and functioning questionnaire)	53/415 (12.8%)
Ding (2025) (23)	China	Cross-sectional study	Patients with maintenance hemodialysis treated for more than 3 months, ≥18 years old	The hemodialysis centers of three hospitals	Cognitive impairment (MoCA scale)	211/276 (76.4%)
Qin (2025) (20)	China	Explorative cross-sectional study	Patients with maintenance hemodialysis treated for 3 months, 18–85 years old	Five hemodialysis centers in two hospitals	Cognitive frailty (FP and MMSE scale)	87/496 (17.5%)
Song (2025) (29)	China	Cross-sectional study	Patients with hemodialysis treated for 3 months, ≥18 years old	The nephrology ward of a hospital	Cognitive impairment (MoCA scale)	134/172 (77.9%)
Shao (2024) (25)	China	Prospective cohort study	Patients with peritoneal dialysis treated for more than 3 months, 18–75 years old	The nephrology ward of a hospital	Cognitive impairment (MoCA scale)	68/120 (56.7%)
Wei (2024) (26)	China	Prospective cohort study	Patients with maintenance hemodialysis treated for more than 3 months, 18–70 years old	A hospital	Cognitive frailty (FP, CDR, and MMSE scale)	131/507 (25.8%)
Xu (2024) (11)	China	Cross-sectional survey	Patients with non-dialysis chronic kidney disease, ≥18 years old	The nephrology outpatient clinics of two hospitals	Mild cognitive impairment (MoCA and MMSE scale)	192/383 (50.1%)
Yang (2024) (10)	China	Cross-sectional survey	Patients with non-dialysis chronic kidney disease, ≥18 years old	The nephrology department in two tertiary healthcare facilities	Mild cognitive impairment (MoCA and MMSE scale)	210/416 (50.9%)
Yi (2024) (27)	China	Prospective cohort study	Patients with maintenance hemodialysis treated for more than 3 months, ≥45 years old	The nephrology ward of a hospital	Cognitive frailty (MoCA, FP, and CDR scale)	43/258 (16.7%)
Zhou (2024) (22)	China	Retrospective study	Patients with chronic kidney disease, ≥60 years old	NHANES data from the CDC of U. S.	Cognitive impairment (cognitive test)	146/545 (26.8%)
Chen (2023) (18)	China	Retrospective study	Patients with maintenance hemodialysis treated for 3 months, ≥18 years old	A hospital	Cognitive impairment (MoCA scale)	46/146 (31.5%)
Fan (2023) (12)	China	Prospective cohort study	Patients with maintenance hemodialysis treated for more than 3 months, >18 years old	A hospital	Cognitive impairment (MoCA scale)	62/100 (62.0%)
Jiang (2023) (24)	China	Cross-sectional study	Patients with maintenance hemodialysis treated for more than 6 months, >18 years old	A hospital	Cognitive impairment (MoCA scale)	105/160 (65.6%)
Sun (2023) (21)	China	Cross-sectional survey	Patients with maintenance hemodialysis treated for more than 6 months, 18–60 years old	A hospital	Cognitive impairment (MoCA scale)	30/50 (60.0%)
Chang (2022) (9)	China	Cross-sectional survey	Patients with chronic kidney disease for more than 3 months, ≥60 years old	The nephrology department of a hospital	Cognitive frailty (MMSE and Frail scale)	154/1015 (15.2%)
Luo (2022) (19)	China	Cross-sectional survey	Patients with chronic kidney disease, ≥60 years old	Two hospitals in southern China	Cognitive frailty (FP and MMSE scale)	93/425 (21.9%)

MoCA, the Montreal cognitive assessment; FP, frailty phenotype; CDR, clinical dementia rating; MMSE, mini-mental state examination.

The main outcome comprised three categories: CI, mild CI, and cognitive frailty. CI was defined as a decline in cognitive function detected by validated screening instruments, without further specification of subtype or dementia exclusion criteria. The commonly used tools were the Montreal Cognitive Assessment (MoCA) and the Mini-Mental State Examination (MMSE). Mild CI was defined as objective CI in one or more domains, preservation of activities of daily living, and absence of dementia (30). All mild CI models used both MoCA and MMSE in combination (10, 11) in the CKD population. Cognitive frailty was defined as the simultaneous presence of physical frailty and CI, excluding dementia (31). Physical frailty was assessed using the Frailty Phenotype scale or the FRAIL scale; CI was assessed using MMSE or MoCA, and dementia was excluded using the Clinical Dementia Rating scale or by clinical judgment.

### 3.3 Overview of model information

Table 2 shows the model information in the included studies. Only 7 studies reported the methods for handling missing data or the reasons for missing data (8, 10, 11, 20–22, 28); Five studies directly excluded cases with missing data (10, 11, 20–22), and multiple interpolation was applied to 2 studies (8, 28). Nine studies did not report information on missing data (9, 18, 19, 23–27, 29). As for the way of variable selection before modeling, the most commonly used method was univariate analysis (*n* = 14, 82.4%) (8–11, 18, 19, 21–28); 3 models used the shrinkage techniques for variable selection (12, 20, 29). Multivariate logistic regression analysis was applied to develop prediction models in most studies (*n* = 13, 76.5%) (9–12, 18, 20, 22–27, 29). Machine learning algorithms served as base classifiers to identify the optimal ensemble model (8, 28), and other studies utilized back propagation neural network (21) and artificial neural network methods (19). Most studies reported the model discrimination assessed using the AUC or C-statistic (*n* = 14, 82.4%). Calibration was reported in 12 models (8–12, 20, 22, 24–28), with the Hosmer–Lemeshow test, calibration plot, or Brier score. Fifteen studies performed internal validation (8–12, 19–28), and seven studies performed external validation (8, 11, 12, 21, 22, 24, 26). Only two studies (8, 22) used truly independent cohorts (cross-regional data or national database). Three studies used validation cohorts from the same hospital (12, 16, 24), 1 study from different campuses of the same hospital (21), and 1 study from different hospitals within the same city (11), which limits the ability to adequately test generalizability across diverse healthcare settings.

**Table 2:** Overview of the information on the included prediction models.

Author (year)	Missing data handling	Variable selection prior to modeling	Predictor selection in modeling	Modeling method	Model validation	Sample size of external validation	Final predictors	Discrimination	Calibration	Other indexes	Model presentation
Cao (2025) (8)	Multiple imputation	Univariate analysis	Backward stepwise selection	Machine learning techniques	Internal validation (5-fold cross validation) and external validation	269	Age, mode of residence, medical payment method, exercise, alcohol consumption, dialysis vascular access, serum albumin classification, serum phosphorus classification, total cholesterol classification, blood urea nitrogen classification, malnutrition score, and depression score	Int: AUC: 0.911 (0.8653, 0.9494); Ac: 0.903; Pr: 0.912; Re: 0.983; F1 score: 0.946 Ext: AUC: 0.832	Calibration plot	DCA: better benefit	Models on web platforms
Cao (2025) (28)	Multiple imputation	Univariate analysis	Backward stepwise selection	Machine learning techniques	Internal validation	Training and test sets (75–25 ratio)	Age, education level, and hemoglobin concentration	Int: AUC: 0.918 (0.8288, 0.977); Ac: 0.808 (0.7307, 0.8846); Re: 0.941 (0.8, 1.0); Sp: 0.782 (0.6962, 0.8681); Pr: 0.457 (0.2972, 0.6285); F1 score: 0.615 (0.439, 0.7636)	Calibration plot	–	Optimal model on the web page
Ding (2025) (23)	–	Univariate analysis	Backward stepwise selection	Logistic regression	Internal validation	–	Age, sleep disorders, years of education, levels of α-Klotho, and levels of β-Klotho	Int: AUC: 0.894 (0.766, 0.887); Se: 82.9%; Sp: 78.5%	–	–	Nomogram
Qin (2025) (20)	Complete-case analysis	Shrinkage techniques (LASSO)	Backward stepwise selection	Logistic regression	Internal validation (bootstrap resampling)	–	Health empowerment, alexithymia, age, educational level, marital status and dialysis vintage	Int: AUC: 0.917 (0.881, 0.956); Se: 85.1%; Sp: 87.8%	Calibration plot and H–L test	DCA: benefit (>0.189)	Nomogram
Song (2025) (29)	No missing data	Shrinkage techniques (LASSO)	Backward stepwise selection	Logistic regression	–	–	Gender, age, protein intake, and cholesterol	–	–	Nagelkerke *R*^2^ = 0.543	Formula
Shao (2024) (25)	–	Univariate analysis	Backward stepwise selection	Logistic regression	Internal validation	–	Diabetes, high levels of glycosylated hemoglobin, parathyroid hormone, and serum ferritin	–	Calibration plot	DCA: positive benefit (0.2–0.8)	Nomogram
Wei (2024) (26)	–	Univariate analysis	Backward stepwise selection	Logistic regression	Internal validation and external validation	223	Age, self-care ability, educational level, diabetes mellitus, hypertension, depression, sleep quality, social support, serum albumin, C-reactive protein, hemoglobin, blood creatinine, triglycerides, total cholesterol, low-density lipoprotein	Int: AUC: 0.945 (0.910, 0.967); Se: 97.14%; Sp: 93.46% Ext: AUC: 0.904 (0.901, 0.938); Se: 88.61%; Sp: 97.22%	Calibration plot	–	–
Xu (2024) (11)	Complete-case analysis	Univariate analysis	Backward stepwise selection	Logistic regression	Internal validation (bootstrap resampling) and external validation	137	Age, educational level, occupation status, use of smartphone, sleep disorders, hemoglobin, and platelet count	Int: AUC: 0.928 (0.902, 0.953) Ext: AUC: 0.897 (0.844, 0.950) ^*^Not labeled: Se: 0.853; Sp: 0.854; Ac:0.854; Re:0.853; Pr: 0.853; F1 score: 0.853; PPV:0.853; NPV:0.854	Calibration plot and H–L test	DCA: benefit (18–95%)	A nomogram and an online calculator
Yang (2024) (10)	Complete-case analysis	Univariate analysis	Backward stepwise selection	Logistic regression	Internal validation (bootstrap resampling)	–	Age, educational level, occupational status, use of smartphones, sleep disorder, and hemoglobin	Int: AUC: 0.926 (0.925, 0.927); Se: 0.767; Sp: 0.932; Ac: 0.849; F1 score: 0.836; PPV: 0.920	Calibration plot and H–L test	DCA: benefit (12.5–98%)	Nomogram
Yi (2024) (27)	–	Univariate analysis	Backward stepwise selection	Logistic regression	Internal validation	–	The calf muscle circumference, five- times-sit-to-stand test, the sarcopenia screening scale score, and the geriatric depression scale score	Int: AUC: 0.842 (0.779, 0.905) Se: 72.1%; Sp: 85.6%	Calibration plot and H–L test	–	Nomogram
Zhou (2024) (22)	Complete-case analysis	Univariate analysis	Backward stepwise selection	Logistic regression	Internal validation (5-fold cross validation) and external validation	131	Age, race, education, annual family income, body mass index, estimated glomerular filtration rate, serum albumin, and uric acid	Int: C-statistic: 0.764 (0.763, 0.807) Ext: C-statistic: 0.752 (0.654, 0.850)	Brier score	Kaplan–Meier survival: log-rank, *p* = 0.00042	Nomogram
Chen (2023) (18)	–	Univariate analysis	Backward stepwise selection	Logistic regression	–	–	Age (aged 55.0–64.0 years, aged 65.0–74.0 years, aged ≥75.0 years), duration of dialysis (≥5 years), and current smoker	Int: AUC: 0.84 (0.77, 0.91)	–	–	–
Fan (2023) (12)	No missing data	Shrinkage techniques (LASSO)	–	Logistic regression	Internal validation and external validation	71	Age, dialysis vintage, higher sensitive C-reactive protein, and lower albumin	Int: AUC: 0.892 (0.813, 0.945); Se: 75.81%; Sp: 89.47% Ext: AUC: 0.899 (0.804, 0.958); Se: 75.00%; Sp: 96.30%	Calibration plot	–	–
Jiang (2023) (24)	–	Univariate analysis	Backward stepwise selection	Logistic regression	Internal validation and external validation	80	Age (>64 years old), disease duration (>4 years), education level (junior high school and below), anxiety, depression, hemoglobin (>102.74 g/L), dialysis duration (>31 months), and care method (routine care)	Int: C-statistics: 0.745 (0.647, 0.811) Ext: AUC: 0.895 (0.816, 0.975); Se: 80.77% Sp: 89.29%	Calibration plot and H–L test	DCA: benefit (>0.07)	Nomogram
Sun (2023) (21)	Complete-case analysis	Univariate analysis	–	Back propagation neural network (BPNN)	Internal validation and external validation	15	Hemoglobin, urea nitrogen, and mean low-frequency amplitude value in the left central posterior gyrus	–	–	*R*^2^_external_: 0.7328 MSE _external_: 0.5007 RMSE _external_: 2.7407 MAE _external_: 2.4246 MAPE _external_: 15.54%	–
Chang (2022) (9)	–	Univariate analysis	Backward stepwise selection	Logistic regression	Internal validation	–	Advanced age, depression, low social support, Charlson comorbidity index, eGFR, and albuminuria	Int: AUC: 0.91 (0.89, 0.94)	Calibration plot	–	Nomogram
Luo (2022) (19)	–	Univariate analysis	Backward stepwise selection	Artificial neural network model (ANN)	Internal validation (random split of data)	–	Barthel Index score, albumin, education level, 15-item geriatric depression scale score, and social support rating scale score	Int: AUC: 0.913; Ac: 86.36%; Sp: 88.61%; Se: 80.65%	–	–	–

“–”, not reported; Cal, calibration; Disc, discrimination; Int; internal validation; Ext, external validation; H–L test, Hosmer–Lemeshow test; DCA, decision curve analysis; Se, sensitivity; Sp, specificity; Ac, accuracy; Pr, precision; Re, recall; PPV, positive predictive value; NPV, negative predictive value; BS, Brier score. ^*^Not labeled as internal or external.

### 3.4 Risk of bias and applicability

The results of ROB and applicability for the included models were summarized in Table 3. Overall, all included studies were assessed as at high ROB, indicating methodological concerns during the development or validation processes. In the participant domain, 5 studies were rated as a source of high ROB because inappropriate data sources were used (18, 19, 22, 24, 28). As for the predictor domain, 14 studies had an unclear ROB. One study did not provide the definition or assessment of predictors (21); 13 studies failed to report information on blinded assessment of predictors before outcome evaluation (8–11, 18, 19, 22, 23, 25–29). Regarding the outcome domain, two studies were at high ROB; a study did not exclude predictors from the outcome definition (9), and a study failed to ensure an appropriate time interval between outcome determination and predictor assessment (24). The blinded assessments between outcomes and predictors were unclear in 16 studies (8–12, 18, 19, 21–29). Thirteen studies lacked information on the timing of the assessment of predictors and outcomes (8–11, 18–22, 25–28). As for the Analysis domain, all included studies were rated as having a high ROB. Most of the studies had insufficient sample sizes; in 6 studies (12, 21, 24, 26, 28, 29), the events per variable (EPV) were less than 10, and in 8 studies (8, 18–20, 22, 23, 25, 27), the EPV was between 10 and 20. Six studies converted continuous variables into categorical variables (10, 11, 18, 22, 24, 26). Four studies excluded missing data (10, 11, 21, 22), two of which excluded variables with more than 10% missing data (10, 11). Besides, sensitivity analysis showed no effect on the results though missing data were excluded in the study of Qin et al. (20). Eight studies did not provide information on missing data (9, 18, 19, 23–27). Fourteen studies employed univariate analysis to screen variables before modeling (8–11, 18, 19, 21–28). A study lacked the assessment of model discrimination performance (25), and three studies failed to assess the model calibration (18, 19, 23). Neither the discrimination nor the calibration of the prediction model was considered in the two studies (21, 29). Ten studies failed to address potential issues of overfitting, optimism, or underfitting in model performance (12, 18, 20, 21, 23–27, 29). One study applied these rounded number scores coming from the derivative of the original weights of the predictors (18). Two studies lacked information on the predictor coefficients in the multivariate regression models (19, 21). No information pertaining to complexities in the data was provided in all included studies.

**Table 3:** PROBAST results of the included studies.

Author (year)	ROB				Applicability			Overall	
	Participants	Predictors	Outcome	Analysis	Participants	Predictors	Outcome	ROB	Applicability
Cao (2025) (8)	+	?	?	−	−	+	−	−	−
Cao (2025) (28)	−	?	?	−	−	+	+	−	−
Ding (2025) (23)	+	?	?	−	−	+	+	−	−
Qin (2025) (20)	+	+	?	−	−	+	−	−	−
Song (2025) (29)	+	?	?	−	+	+	+	−	+
Shao (2024) (25)	+	?	?	−	−	+	+	−	−
Wei (2024) (26)	+	?	?	−	−	+	−	−	−
Xu (2024) (11)	+	?	?	−	+	+	+	−	+
Yang (2024) (10)	+	?	?	−	+	+	+	−	+
Yi (2024) (27)	+	?	?	−	−	+	−	−	−
Zhou (2024) (22)	−	?	?	−	+	−	+	−	−
Chen (2023) (18)	−	?	?	−	−	+	+	−	−
Fan (2023) (12)	+	+	?	−	−	+	+	−	−
Jiang (2023) (24)	−	+	−	−	+	+	+	−	+
Sun (2023) (21)	+	?	?	−	−	?	+	−	−
Chang (2022) (9)	+	?	−	−	−	+	−	−	−
Luo (2022) (19)	−	?	?	−	−	+	−	−	−

ROB, risk of bias; +, indicates low ROB/low concern regarding applicability; −, indicates high ROB/high concern regarding application; ?, indicates unclear ROB/unclear concern regarding applicability.

For the applicability evaluation, 13 studies were considered at high concern regarding application (8, 9, 12, 18–23, 25–28). Regarding participants’ domain, 12 studies were assessed to have great concerns because participants were limited to specific subgroups of dialysis or older adults patients (8, 9, 12, 18–21, 23, 25–28). In the predictor domain, one study was deemed at high risk because of the concerns regarding the timing of predictor measurement (22). One study was judged to be at unclear risk as no reporting regarding the definition and evaluation of predictors (21). In the outcome domain, 6 studies had great concerns of applicability for the outcomes included not only CI but also frailty (8, 9, 19, 20, 26, 27). Besides, the evidence is largely derived from Chinese populations (94.1%), limiting generalizability to other ethnic groups. The certainty of evidence for non-Chinese settings was decreased. Details of the quality assessment in each study are shown in Supplementary Table 2.

### 3.5 Subgroup analyses

To explore sources of heterogeneity, we performed subgroup analyses stratified by outcome definitions, CKD population, and cognitive assessment tools. Due to the heterogeneity in outcomes, populations, and assessment tools, as well as the high risk of bias in all studies, no direct or indirect comparison of model performance was conducted.

#### 3.5.1 Subgroup by outcome definitions

(1) Model performance and validation

As shown in Table 4 and Supplementary Table 3, CI was reported in 9 models (12, 18, 21–25, 28, 29). The reported prevalence of CI varied from 12.8 to 77.9%. Internal validation was performed in 8 models (88.9%), with AUC ranging from 0.745 to 0.918. External validation was performed in 4 models (44.4%), with AUC ranging from 0.752 to 0.899. Calibration was reported in 5 models (55.6%), using calibration plots (*n* = 4), Hosmer–Lemeshow test (*n* = 1), or Brier score (*n* = 1). DCA was reported in 2 models, both showing net benefit.

**Table 4:** Summary of model performance, validation, and risk of bias by outcomes (*n* = 17).

Outcome	Models (*n*)	Internal validation (*n*)	External validation (*n*)	AUC	Calibration (*n*)	High ROB (*n*)
CI	9	8	4	Int: 0.745–0.918 Ext: 0.752–0.899	5	9
CF	6	6	2	Int: 0.842–0.945 Ext: 0.832; 0.904	5	6
MCI	2	2	1	Int: 0.926; 0.928 Ext: 0.897	2	2

CI, cognitive impairment; CF, cognitive frailty; MCI, Mild cognitive impairment; Int, internal validation; Ext, external validation.

Mild CI was reported in 2 models (10, 11). The reported prevalence of Mild CI was 50.1 and 50.9%, respectively. Both models had internal validation, with AUCs of 0.926 and 0.928, respectively, and one had external validation (AUC: 0.897). Calibration was reported in both models (Hosmer–Lemeshow test and calibration plots). DCA was reported in both models, indicating clinical benefit.

Cognitive frailty was reported in 6 models (8, 9, 19, 20, 26, 27). The reported prevalence of CI varied from 14.2 to 25.8%. All models underwent internal validation, with AUC ranging from 0.842 to 0.945. External validation was performed in 2 models (33.3%), with AUC of 0.832 and 0.904, respectively. Calibration was reported in 5 models (83.3%), mainly using calibration plots (*n* = 5) and the Hosmer–Lemeshow test (*n* = 2). DCA was reported in 2 models (33.3%), showing positive net benefit.

(2) Predictors

**Figure 2:**
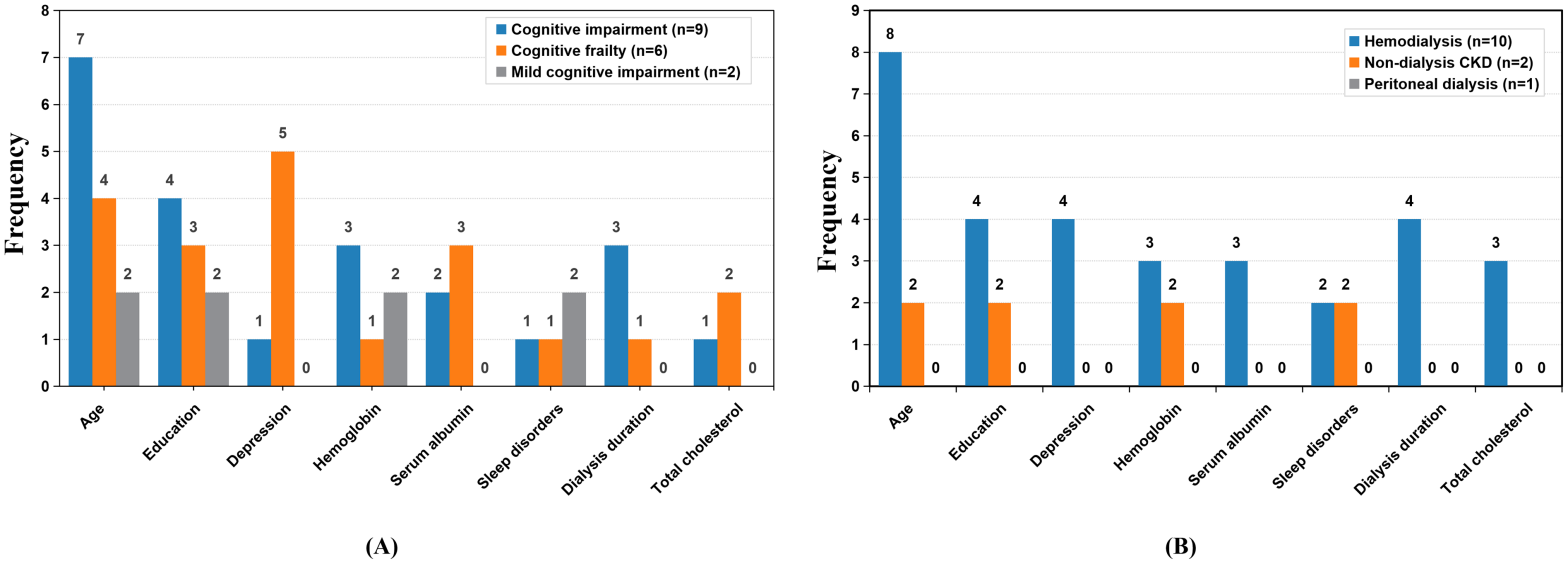
Frequency of predictors across subgroups stratified by outcome definitions and CKD population (total frequency ≥3). **(A)** Outcome definitions; **(B)** CKD population.

Age was the most frequent predictor across all outcome groups. Education was also common. Depression was reported in 83.3% of cognitive frailty models, in 11.1% of unspecified CI models, and was not reported in mild CI models. Hemoglobin appeared in all mild CI models and in 3 CI models (33.3%) but only in 1 cognitive frailty model (16.7%). Serum albumin was reported in half of the cognitive frailty models and in 2 CI models (22.2%). The details of the predictors were shown in Figure 2A.

#### 3.5.2 Subgroup by CKD population

(1) Model performance and validation

As shown in Table 5 and Supplementary Table 4, hemodialysis was reported in 10 models (8, 12, 18, 20, 21, 23, 24, 26, 27, 29). Among these models, 8 models had internal validation (80.0%), with AUC ranging from 0.745 to 0.945. External validation was performed in 4 models (40.0%), with AUC from 0.832 to 0.904. Calibration was reported in 6 models (60%), mainly using calibration plots (*n* = 6) and the Hosmer–Lemeshow test (*n* = 3). DCA was reported in 3 models (30.0%), both showing net benefit.

**Table 5:** Summary of model performance, validation, and risk of bias by CKD population (*n* = 13).

CKD population	Models (*n*)	Internal validation (*n*)	External validation (*n*)	AUC	Calibration (*n*)	High ROB (*n*)
Hemodialysis	10	8	4	Int: 0.745–0.945 Ext: 0.832–0.904	6	10
Peritoneal dialysis	1	1	0	–	1	1
Non-dialysis CKD	2	2	1	Int: 0.926; 0.928 Ext: 0.897	2	2

“–”, not reported; Int, internal validation; Ext, external validation.

Peritoneal dialysis was reported in only 1 model (25). This model did not report discrimination but did report calibration (calibration plot). No external validation or DCA was performed. Due to the single model, no meaningful subgroup summary can be made. Findings from this subgroup should be considered hypothesis-generating only.

Non-dialysis CKD was reported in 2 models (10, 11). Both models had internal validation, with AUCs of 0.928 and 0.926, respectively. External validation was performed in one model (50.0%), with an AUC of 0.897. Calibration was reported in 2 models (100.0%), using the Hosmer–Lemeshow test and calibration plots. DCA was reported in 2 models (100.0%), all showing net benefit.

Besides, four models were developed for CKD populations, but the specific CKD stages were not specified (9, 19, 22, 28), so a narrative synthesis was not performed.

(2) Predictors

As shown in Figure 2B, age was the most frequent predictor in both hemodialysis (80.0%) and non-dialysis CKD (100.0%) groups. Education appeared in 4 hemodialysis models (40.0%) and 2 non-dialysis CKD models (83.3%). Hemoglobin occurred in 3 hemodialysis (30.0%) and 2 non-dialysis CKD models (100.0%). Depression, dialysis duration (40.0%), serum albumin (30.0%), and total cholesterol (30.0%) were reported only in hemodialysis models. The single peritoneal dialysis model included diabetes, HbA1c, parathyroid hormone, and ferritin. None of the included models considered nephrology parameters such as dialysis dose, ultrafiltration rate, intradialytic hypotension, dialyzer type, or dialysate composition.

#### 3.5.3 Subgroup by cognitive assessment tools

Among the 17 models, nine models used the MoCA to assess cognitive function (8, 12, 18, 21, 23–25, 27, 29), and four used the MMSE (9, 19, 20, 26). Besides, two studies used both MoCA and MMSE to evaluate mild CI (10, 11). MoCA was predominant in hemodialysis models (80.0%). The internal AUC of the MoCA-based models ranged from 0.745 to 0.911, while that of the MMSE-based models ranged from 0.91 to 0.945. External validation was performed in only one MMSE-based model (AUC: 0.904) and in 4 MoCA-based models (44.4%) with AUC from 0.832 to 0.899. Calibration was reported in 5 MoCA-based models (55.6%) and 3 MMSE-based models (75.0%). DCA was reported in 3 MoCA-based models (33.3%) and 1 MMSE-based model (25.0%). Two models using both MoCA and MMSE reported the internal AUC of 0.926 and 0.928; one performed external validation (AUC: 0.897) (Table 6 and Supplementary Table 5).

**Table 6:** Summary of model performance, validation, and risk of bias by cognitive assessment tools (*n* = 15).

Cognitive assessment tools	Models (*n*)	Internal validation (*n*)	External validation (*n*)	AUC	Calibration (*n*)	High ROB (*n*)
MoCA	9	6	3	Int: 0.745–0.911 Ext: 0.832–0.899	5	9
MMSE	4	4	1	Int: 0.91–0.945 Ext: 0.904	3	4
MoCA and MMSE	2	2	1	Int: 0.926; 0.928 Ext: 0.897	2	2

MoCA, the Montreal cognitive assessment; MMSE, mini-mental state examination; Int, internal validation; Ext, external validation.

## 4 Discussion

Effectively assessing the potential risk of CI in patients with CKD for early identification and intervention is essential, as it may significantly reduce adverse outcomes (10). This is the first systematic review to synthesize and reveal critical methodological gaps of existing prediction models for CI in patients with CKD. A total of 17 studies and corresponding models were included, summarizing the performance and characteristics of models, while also assessing the ROB and applicability. According to the PROBAST assessment, all models demonstrated high ROB, and 13 models showed great concerns regarding applicability; the availability of prediction models was limited. Subgroup analyses stratified by outcome definitions, CKD population, and cognitive assessment tools showed considerable variation in reported prevalence and AUC values across subgroups. Current model development studies exhibit limited sample sizes, with merely two investigations utilizing large-scale cohorts (over 1,000) for prediction modeling (8, 9). Most studies were based on data from Chinese patients. This limits generalizability to other ethnic groups, healthcare systems, and the CKD population. The single NHANES-based model partially addresses this, but evidence for non-Chinese settings remains insufficient. So the certainty of evidence for populations outside China is downgraded to very low. Consequently, future research necessitates the development of prediction models rigorous, utilizing multicenter external validation, larger cohorts, and enhanced reporting transparency, such as adhering to TRIPOD guidelines (32).

The prevalence of CI varied considerably across the included studies, ranging from 12.8 to 77.9%. Some studies focused on cognitive frailty in CKD patients, a syndrome defined by the co-existence of physical frailty and mild CI while excluding Alzheimer’s disease and other forms of dementia (33). Subgroup analyses stratified by outcome definitions showed that prevalence was reported as 12.8–77.9% for unspecified CI, approximately 50% for mild CI, and 14.2–25.8% for cognitive frailty. This variation reflects differences in outcome definitions, participant characteristics, and cognitive assessment tools. Current prediction models target CKD patients at different disease stages, including non-dialysis CKD, hemodialysis, and peritoneal dialysis, as well as different age groups. Notably, cognitive decline was significantly associated with impaired kidney function and advanced age (34). Cognitive function declines even in the early stages of CKD (Stages 1–2); Seliger et al. (35) demonstrated that moderate kidney impairment is strongly associated with an increased risk of dementia. Furthermore, some studies have reported a higher prevalence of CI (mainly manifested as impaired executive function) in hemodialysis patients (36), while Kalirao et al. (37) suggested that severe CI was more common in peritoneal dialysis patients compared to those on hemodialysis. The findings highlight the further exploration of the characteristics and progression patterns of CI across different stages of CKD, which would facilitate early identification and targeted interventions. This review also identified marked variation in cognitive assessment tools (MoCA, MMSE, health questionnaires, and cognitive tests), which may contribute to the observed heterogeneity in reported prevalence and model discrimination.

The frequency of predictors varied across subgroups defined by outcome and CKD population. Across all 17 models, age and education were the most frequently reported predictors. Age and education were frequently reported across all subgroups. The biological plausibility of age as a predictor is well established. Shang et al. (38) revealed a significantly increased risk of CI in middle-aged populations with CKD. Yang et al. (10) reported that CKD patients over 65 or older had a 2.941-fold higher risk of developing mild CI compared to those under 65 years of age. With advancing age, natural cognitive decline occurred alongside increased vulnerability to cardiovascular risk factors, which caused cerebrovascular pathology and subsequent CI (39, 40). Higher education level served as a protective factor for cognitive function, which was probably attributable to sustained stimulation and training of brain cells, effectively enhancing cerebral functioning and mitigating CI progression (41). Depression was identified as a common predictor, particularly in cognitive frailty models. However, the interpretation of this association is complicated by several factors. Depression was associated with pathological factors including chronic inflammation, oxidative stress, and cerebral white matter lesions; it manifested clinically as slowed cognitive processing speed, reduced volitional activity, and diminished social interaction (8, 42). The symptom profiles of depression and early CI overlap substantially (psychomotor slowing, reduced concentration.) (43). This clinical overlap can lead to pseudodementia and may inflate the predictive value of depression when cognitive outcomes are assessed concurrently. Most included studies did not specify whether depression assessment preceded cognitive outcome evaluation. Due to the lack of a clear temporal sequence, reverse causality is unclear: while depression might contribute to CI, CI could also precipitate or worsen depressive symptoms through loss of independence, social withdrawal, and perceived decline (44). Therefore, although depression emerged as a frequent predictor in the reviewed models, the independent contribution of depression to incident CI remains uncertain. Strikingly, none of the models incorporated key nephrology parameters (ultrafiltration rate, dialysis dose, intradialytic hypotension.), despite accumulating evidence of their importance for cognitive outcomes. In a prospective cohort of 121 older haemodialysis patients, cumulative ultrafiltration volume significantly correlated with a reduction in cerebral arterial mean flow velocity, and repetitive intradialytic decreases in cerebral blood flow may be one of the mechanisms underlying the decline of cognitive function (45). It’s reported that maintenance hemodialysis patients with recurrent intradialytic hypotension exhibit significant declines in cognitive function (particularly executive function and attention) and emotional status (46). It’s a notable gap given these modifiable factors to cerebral perfusion and cognitive outcomes. Integrating such parameters into future prediction models represents an important opportunity to develop clinically actionable tools for personalized management and cognitive protection among CKD patients. Furthermore, certain laboratory indicators included in the prediction models (such as C-reactive protein and hemoglobin), and their clinical interpretability is limited by the malnutrition-inflammation complex syndrome (MICS) as a major confounder, unexamined threshold effects, and time-varying bias due to disease progression and treatment. Static single-point measurements are clinically insufficient for dynamic decision making. Future models should prioritize repeated longitudinal measurements and explicitly account for MICS.

A critical finding of this review is that all included studies were assessed as having a high overall risk of bias, predominantly stemming from the analysis domain. Inappropriate methods were employed for handling missing data, which potentially introduced selection bias. Four studies directly excluded cases with missing data, and eight studies did not report any information on missing data handling. Such approaches, particularly complete-case analysis, not only reduce statistical power but also risk selection bias, ultimately limiting the model’s applicability to real-world populations where missing data are routine. Consequently, these models should not be recommended for clinical practice without rigorous external validation and missing data sensitivity analyses. Regarding predictor selection, univariate analysis was the most commonly used approach, a practice that ignores the complex interplay between candidate variables and can introduce bias, discouraged in modern predictive modeling guidelines (47). In contrast, three studies used LASSO regression for variable selection (12, 20, 29), a method that not only identified optimal predictors but also mitigated overfitting (48). Besides, several included studies had insufficient sample sizes for model development. Most of the studies had fewer than 20 EPV. This prevalent issue raises serious concerns about model overfitting, potentially leading to overly optimistic performance estimates that may not replicate in new settings. This may reduce statistical power, increase the risk of Type II errors, have poor generalization to different populations, and limit clinical applicability (47). Furthermore, this systematic review revealed significant limitations in the validation and evaluation of prediction models for CI in CKD patients. Although 15 models underwent some form of validation, only 7 performed both internal and external validation. External validation remains superficial. Among 7 models, 5 used validation cohorts derived from the same hospital, different campuses of the same hospital, or different hospitals within the same region, which cannot be considered truly independent for assessing generalizability across diverse healthcare settings. Future research must prioritize truly independent, multi-center, cross-regional external validation with explicit cohort comparability analyses. Calibration was also inadequately assessed: 6 studies lacked calibration evaluation entirely. The paucity of external validation and calibration evaluation may limit the applicability of these prediction models in the real world (49). Therefore, it is suggested to improve the overall quality of the research on CI risk prediction model studies. Methodological limitations in existing predictive model research should be addressed, and the development and validation of the prediction model for CI in CKD patients should be conducted in accordance with TRIPOD (32), PROBAST, and the step-by-step guidelines (47). Notably, machine learning methods are recommended for handling sample size, variable selection, and model development. Compared to traditional logistic regression, machine learning methods tend to have superior accuracy (50), but the interpretability and presentation are limited. The SHapley Additive exPlanations tool was employed to visualize the decision-making processes of ML models (51). Thus, the appropriate modeling approach should be determined according to specific application scenarios and practical needs.

The existing prediction models are characterized by substantial heterogeneity in the choice of cognitive assessment tools. The included studies used MoCA, MMSE, health status questionnaires, and general cognitive tests, which have different sensitivities, specificities, and domain coverage. In CKD patients, executive dysfunction is typical (36). The MMSE tool, which primarily assesses memory, may underestimate the true prevalence of CI, while the MoCA tool has greater sensitivity to executive and attentional domains. However, even among MoCA-based models in this review, different versions and cut-off values were used, further limiting comparability. The single study that used a non-validated health questionnaire (28) or a general NHANES cognitive test (22) cannot be directly compared with those using standardized instruments. Consequently, the reported performance metrics may not be directly transferable across studies that used different tools. Future prediction model studies should adopt a unified and validated cognitive assessment tool, with standardized administration and cut-offs. If multiple tools are unavoidable, separate model development and validation for each tool should be considered, and the tool’s domain sensitivity should be explicitly discussed. A further critical finding identified by this review is the absence of cognitive domain-specific prediction models. All models included treated CI as a global and binary outcome, without distinguishing between distinct cognitive domains such as executive function, memory, or attention. CKD, particularly end-stage renal disease, is known to disproportionately affect executive function compared to other domains (36), which has direct implications for medication adherence, self-care, and dietary management. Therefore, we strongly emphasize that developing and validating prediction models for domain-specific CI, particularly executive dysfunction. Future studies should consider using domain-specific neuropsychological batteries combined with global screening instruments for cognitive function. The cognitive domain-specific models probably support tailored cognitive rehabilitation and improve real-world functional outcomes in CKD populations.

### 4.1 Implications for public health

CI in CKD remains a largely underrecognized public health challenge associated with increased healthcare utilization, frailty, and reduced quality of life. This study identifies several implications for public health practice and population health management, while also highlighting important limitations that must be addressed before clinical application. This review highlights specific priorities for cognitive risk early stratification among CKD: (1) adoption of standardized outcome definitions and validated assessment tools; (2) large-scale, multi-center, and cross-regional external validation studies; (33) development of prediction models targeting domain-specific impairments such as executive dysfunction, which is common in CKD but currently ignored. By systematically exposing these methodological gaps, this review provides an evidence-based roadmap for future public health research aimed at improving cognitive risk stratification in CKD. These findings can inform public mental health strategies, aging-related health policies, and integrated care pathways for patients with CKD. Through improving the early identification of high-risk individuals, this work helps reduce the public health burden of CI and promotes equitable, proactive care for vulnerable populations.

### 4.2 Limitations

This study has several limitations. Firstly, the inclusion of publications only in English and the Chinese language might cause the omission of relevant studies, potentially bringing selection bias. Secondly, the lack of large samples and multicenter external validation results had caused certain bias. Besides, due to the heterogeneity in outcomes, populations, and assessment tools, as well as the high risk of bias in all studies, no quantitative synthesis was performed, and no direct or indirect comparison of model performance was conducted. Future research needs to develop more standardized and accurate CI prediction models among CKD populations in different regions, and pay attention to the external generalization of models.

## 5 Conclusion

This systematic review is the first to synthesize existing prediction CI models and reveal critical methodological gaps in CKD. Current prediction models for CI in CKD remain at an early stage. All 17 models have high ROB, predominantly due to methodological shortcomings in analysis, handling of missing data, and variable selection. Outcome definitions and cognitive assessment tools are heterogeneous, preventing direct comparisons or pooling of results. Future modeling should adhere to better transparency of reporting to improve the quality of studies and prioritize larger sample sizes and multicenter external validation across diverse geographic regions.

## Data Availability

The original contributions presented in the study are included in the article/Supplementary material, further inquiries can be directed to the corresponding author.
