# Supplementary material for: Risk prediction models for cognitive impairment in patients with chronic kidney disease: a systematic review
Source: Front Public Health. 2026 Jun 11;14:1851336. doi: 10.3389/fpubh.2026.1851336 (PMC13293797; doi:10.3389/fpubh.2026.1851336)
Supplement: Supplementary file 1 [file Table_1.DOCX]

| **Supplementary Table 1. Search strategy and search terms.** | |
| --- | --- |
| Database | Search terms |
| PubMed | #1 "Renal Insufficiency, Chronic"[Mesh] OR "Renal Dialysis"[Mesh] OR Chronic Kidney Insufficiency[Title/Abstract] OR Chronic Renal Insufficiency[Title/Abstract] OR Chronic Kidney Disease[Title/Abstract] OR Chronic Renal Disease[Title/Abstract] OR chronic kidney disorder[Title/Abstract] OR progressive kidney[Title/Abstract] OR Renal failure[Title/Abstract] OR kidney failure[Title/Abstract] OR End Stage Kidney Disease[Title/Abstract] OR End Stage Renal Disease[Title/Abstract] OR Renal Dialysis[Title/Abstract] OR Hemodialysis[Title/Abstract] OR Peritoneal Dialysis[Title/Abstract] |
|  | #2 Model[Title/Abstract] OR Risk prediction model[Title/Abstract] OR Risk factor[Title/Abstract] OR Predictor[Title/Abstract] OR Risk Score[Title/Abstract] OR risk assessment[Title/Abstract] OR Risk model[Title/Abstract] OR Nomogram[Title/Abstract] |
|  | #3 "Cognitive Dysfunction"[Mesh] OR Cognition[Title/Abstract] OR Cognitive dysfunction[Title/Abstract] OR Cognitive impairment[Title/Abstract] OR Cognitive defect[Title/Abstract] OR Cognitive Disorder[Title/Abstract] OR Cognitive Decline[Title/Abstract] OR Mental Deterioration[Title/Abstract] OR dementia[Title/Abstract] |
|  | #1 AND #2 AND #3 |
| Web of Science | #1 TS=(Renal Insufficiency, Chronic) OR TS=(Chronic Kidney Insufficiency) OR TS=(Chronic Renal Insufficiency) OR TS=(Chronic Kidney Disease) OR TS=(Chronic Renal Disease) OR TS=(chronic kidney disorder) OR TS=(progressive kidney) OR TS=(Renal failure) OR TS=(kidney failure) OR TS=(End Stage Kidney Disease) OR TS=(End Stage Renal Disease) OR TS=(Renal Dialysis) OR TS=(Hemodialysis) OR TS=(Peritoneal Dialysis) |
|  | #2 TS=(Model) OR TS=(Risk prediction model) OR TS=(Risk factor) OR TS=(Predictor) OR TS=(Risk Score) OR TS=(risk assessment) OR TS=(Risk model) OR TS=(Nomogram) |
|  | #3 TS=(Cognitive dysfunction) OR TS=(Cognition) OR TS=(Cognitive impairment) OR TS=(Cognitive defect) OR TS=(Cognitive Disorder) OR TS=(Cognitive Decline) OR TS=(Mental Deterioration) OR TS=(dementia) |
|  | #1 AND #2 AND #3 |
| Embase | #1 ('chronic kidney disease') OR 'renal dialysis':ab,ti OR 'chronic kidney insufficiency':ab,ti OR 'chronic renal insufficiency':ab,ti OR 'chronic kidney disease':ab,ti OR 'chronic renal disease':ab,ti OR 'chronic kidney disorder':ab,ti OR 'progressive kidney':ab,ti OR 'renal failure':ab,ti OR 'kidney failure':ab,ti OR 'end stage kidney disease':ab,ti OR 'end stage renal disease':ab,ti OR 'renal dialysis':ab,ti OR 'hemodialysis':ab,ti OR '[peritoneal dialysis](https://www-ncbi-nlm-nih-gov-s-249.libdb.csu.edu.cn/mesh/68010530)':ab,ti |
|  | #2 ('risk prediction model') OR 'model':ab,ti OR 'risk prediction model':ab,ti OR 'risk factor':ab,ti OR 'predictor':ab,ti OR 'risk score':ab,ti OR 'risk assessment':ab,ti OR 'risk model':ab,ti OR 'nomogram':ab,ti |
|  | #3 ('cognitive dysfunction') OR 'cognition':ab,ti OR 'cognitive dysfunction':ab,ti OR 'cognitive impairment':ab,ti OR 'cognitive defect':ab,ti OR 'cognitive disorder':ab,ti OR 'cognitive decline':ab,ti OR 'mental deterioration':ab,ti OR 'dementia':ab,ti |
|  | #1 AND #2 AND #3 |
| CINAHL | #1 Renal Dialysis OR Chronic Kidney Insufficiency OR Chronic Renal Insufficiency OR Chronic Kidney Disease OR Chronic Renal Disease OR chronic kidney disorder OR progressive kidney OR Renal failure OR kidney failure OR End Stage Kidney Disease OR End Stage Renal Disease OR Renal Dialysis OR Hemodialysis OR [Peritoneal Dialysis](https://www-ncbi-nlm-nih-gov-s-249.libdb.csu.edu.cn/mesh/68010530) |
|  | #2 Model OR Risk prediction model OR Risk factor OR Predictor OR Risk Score OR risk assessment OR Risk model OR Nomogram |
|  | #3 Cognitive dysfunction OR Cognition OR Cognitive impairment OR Cognitive defect OR Cognitive Disorder OR Cognitive Decline OR Mental Deterioration OR dementia |
|  | #1 AND #2 AND #3 |
| Cochrane Library | #1 [mh “renal Insufficiency, chronic”] OR [mh “renal dialysis”] OR (chronic kidney insufficiency):ti,ab,kw OR (chronic renal insufficiency):ti,ab,kw OR (chronic kidney disease):ti,ab,kw OR (chronic renal disease):ti,ab,kw OR (chronic kidney disorder):ti,ab,kw OR (progressive kidney):ti,ab,kw OR (renal failure):ti,ab,kw OR (kidney failure):ti,ab,kw OR (end stage kidney disease):ti,ab,kw OR (end stage renal disease):ti,ab,kw OR (renal dialysis):ti,ab,kw OR (Hemodialysis):ti,ab,kw OR (peritoneal dialysis):ti,ab,kw |
|  | #2 [mh “cognitive dysfunction”] OR cognition:ti,ab,kw OR (cognitive dysfunction):ti,ab,kw OR (cognitive impairment):ti,ab,kw OR (cognitive defect):ti,ab,kw OR (cognitive disorder):ti,ab,kw OR (cognitive decline):ti,ab,kw OR (mental deterioration):ti,ab,kw OR dementia:ti,ab,kw |
|  | #3 model:ti,ab,kw OR (risk prediction model):ti,ab,kw OR (risk factor):ti,ab,kw OR (predictor):ti,ab,kw OR (risk score):ti,ab,kw OR (risk assessment):ti,ab,kw OR (risk model):ti,ab,kw OR nomogram:ti,ab,kw |
|  | #1 AND #2 AND #3 |
| China National Knowledge Infrastructure (CNKI) | #1 SU %= ('慢性肾病' + '慢性肾脏病' + '慢性肾功能不全' + '透析' + '血液透析' + '腹膜透析' + '肾衰竭' + '终末期肾病') |
|  | #2 SU %= ('模型' + '预测模型' + '风险预测' + '危险因素' + '风险因素' + '预测因子' + '列线图' + '风险评分') |
|  | #3 SU %= ('认知障碍' + '认知功能障碍' + '认知衰弱' + '痴呆' + '阿尔兹海默病') |
|  | #1 AND #2 AND #3 [同义词扩展] |
| Wanfang | #1 主题:(慢性肾病 OR 慢性肾脏病 OR 慢性肾功能不全 OR 透析 OR 血液透析 OR 腹膜透析 OR 肾衰竭 OR 终末期肾病) |
|  | #2 主题:(模型 OR 预测模型 OR 风险预测 OR 危险因素 OR 风险因素 OR 预测因子 OR 列线图 OR 风险评分) |
|  | #3 主题:(认知障碍 OR 认知功能障碍 OR 认知衰弱 OR 痴呆 OR 阿尔兹海默病) |
|  | #1 AND #2 AND #3 [主题词扩展] |
| SinoMed | #1 (慢性肾病 OR 慢性肾脏病 OR 慢性肾功能不全 OR 透析 OR 血液透析 OR 腹膜透析 OR 肾衰竭 OR 终末期肾病) OR ("肾功能不全, 慢性"[不加权:扩展]) |
|  | #2 (认知障碍 OR 认知功能障碍 OR 认知衰弱 OR 痴呆 OR 阿尔兹海默病) OR ("认知功能障碍"[不加权:扩展]) OR ("认知障碍"[不加权:扩展]) |
|  | #3 模型 OR 预测模型 OR 风险预测 OR 危险因素 OR 风险因素 OR 预测因子 OR 列线图 OR 风险评分 |
|  | #1 AND #2 AND #3 |
| China Science and Technology Journal Database (VIP) | #1 任意字段: (慢性肾病 OR 慢性肾脏病 OR 慢性肾功能不全 OR 透析 OR 血液透析 OR 腹膜透析 OR 肾衰竭 OR 终末期肾病) |
|  | #2 任意字段: (模型 OR 预测模型 OR 风险预测 OR 危险因素 OR 风险因素 OR 预测因子 OR 列线图 OR 风险评分) |
|  | #3 任意字段: (认知障碍 OR 认知功能障碍 OR 认知衰弱 OR 痴呆 OR 阿尔兹海默病) |
|  | #1 AND #2 AND #3 |

| **Supplementary Table 2. Detailed risk of bias assessment of prediction models for CI in CKD using the PROBAST.** | | | | | | | | | | | | | | | | | | | | |
| --- | --- | --- | --- | --- | --- | --- | --- | --- | --- | --- | --- | --- | --- | --- | --- | --- | --- | --- | --- | --- |
| Author (year) | Participants | | Predictors | | | Outcome | | | | | | Analysis | | | | | | | | |
|  | 1.1 | 1.2 | 2.1 | 2.2 | 2.3 | 3.1 | 3.2 | 3.3 | 3.4 | 3.5 | 3.6 | 4.1 | 4.2 | 4.3 | 4.4 | 4.5 | 4.6 | 4.7 | 4.8 | 4.9 |
| Cao (2025) (8) | Y | Y | Y | NI | Y | Y | Y | PY | Y | NI | NI | PN | Y | Y | Y | N | NI | Y | Y | Y |
| Cao (2025) (28) | N | Y | Y | NI | Y | PY | PY | Y | Y | NI | NI | N | Y | Y | Y | N | NI | Y | Y | Y |
| Ding (20255) (23) | Y | Y | Y | NI | Y | Y | Y | Y | Y | NI | Y | PN | Y | Y | NI | N | NI | N | N | Y |
| Qin (2025) (20) | Y | Y | Y | PY | Y | Y | Y | PY | Y | Y | NI | PN | Y | Y | PY | Y | NI | Y | N | Y |
| Song (2025) (29) | Y | Y | Y | NI | Y | Y | Y | Y | Y | NI | Y | N | Y | Y | Y | Y | NI | N | PN | Y |
| Shao (2024) (25) | Y | Y | Y | NI | Y | Y | Y | Y | Y | NI | NI | PN | Y | Y | NI | N | NI | N | PN | Y |
| Wei (2024) (26) | Y | Y | Y | NI | Y | Y | Y | Y | Y | NI | NI | N | PN | Y | NI | N | NI | Y | PN | Y |
| Xu (2024) (11) | Y | PY | Y | NI | Y | Y | Y | Y | Y | NI | NI | Y | PN | Y | N | N | NI | Y | Y | Y |
| Yang (2024) (10) | Y | Y | Y | NI | Y | Y | Y | Y | Y | NI | NI | Y | PN | Y | N | N | NI | Y | Y | Y |
| Yi (2024) (27) | Y | Y | Y | NI | Y | Y | Y | PY | Y | NI | NI | PN | Y | Y | NI | N | NI | Y | N | Y |
| Zhou (2024) (22) | N | Y | Y | NI | Y | Y | Y | Y | Y | NI | NI | PN | N | Y | N | N | NI | Y | Y | Y |
| Chen (2023) (18) | PN | Y | Y | NI | Y | Y | Y | Y | Y | NI | NI | PN | PN | PY | NI | N | NI | N | PN | PN |
| Fan (2023) (12) | Y | Y | Y | Y | Y | Y | Y | Y | Y | NI | Y | N | Y | Y | Y | Y | NI | Y | PN | Y |
| Jiang (2023) (24) | N | Y | Y | Y | Y | Y | Y | Y | Y | NI | N | N | PN | Y | NI | N | NI | N | PN | Y |
| Sun (2023) (21) | Y | Y | NI | Y | Y | Y | Y | Y | Y | NI | NI | N | Y | Y | N | PN | NI | PN | N | NI |
| Chang (2022) (9) | Y | Y | PY | NI | Y | Y | Y | PN | Y | NI | NI | Y | Y | Y | NI | N | NI | Y | PY | Y |
| Luo (2022) (19) | PN | Y | PY | NI | Y | Y | Y | PY | Y | NI | NI | PN | Y | Y | NI | N | NI | N | Y | NI |
| PROBAST; Prediction model risk of bias assessment tool; Y, Yes; PY, Probably yes; N, No; PN, Probably no; NI, No information.  1.1 Were appropriate data sources used, for example, cohort, RCT, or nested case-control study data? 1.2 Were all inclusions and exclusions of participants appropriate? 2.1 Were predictors defined and assessed in a similar way for all participants? 2.2 Were predictor assessments made without knowledge of outcome data? 2.3 Are all predictors available at the time the model is intended to be used? 3.1 Was the outcome determined appropriately? 3.2 Was a prespecified or standard outcome definition used? 3.3 Were predictors excluded from the outcome definition? 3.4 Was the outcome defined and determined in a similar way for all participants? 3.5 Was the outcome determined without knowledge of predictor information? 3.6 Was the time interval between predictor assessment and outcome determination appropriate? 4.1 Were there a reasonable number of participants with the outcome? 4.2 Were continuous and categorical predictors handled appropriately? 4.3 Were all enrolled participants included in the analysis? 4.4 Were participants with missing data handled appropriately? 4.5 Was selection of predictors based on univariable analysis avoided? (For development model) 4.6 Were complexities in the data accounted for appropriately? 4.7 Were relevant model performance measures evaluated appropriately? 4.8 Were model overfitting, underfitting, and optimism in model performance accounted for? (For development model) 4.9 Do predictors and their assigned weights in the final model correspond to the results from the reported multivariable analysis? (For development model). | | | | | | | | | | | | | | | | | | | | |

| **Supplementary Table 3. The details of model information stratified by Outcome definitions.** | | | | | | | |
| --- | --- | --- | --- | --- | --- | --- | --- |
| Outcomes | Author (year) | Cognitive assessment tools | CKD population | Incidence rate | Final predictors | AUC (95%CI) | Calibration |
| CI | Cao (2025) (28) | Health Status and Functioning Questionnaire | CKD | 12.8% | Age, education level, and hemoglobin concentration | **Int:** 0.918 (0.8288, 0.977) | Calibration plot |
|  | Song (2025) (29) | MoCA | Hemodialysis | 77.9% | Gender, age, protein intake, and cholesterol | -- | -- |
|  | Shao (2024) (25) | MoCA | Peritoneal dialysis | 56.7% | Diabetes, high levels of glycosylated hemoglobin, parathyroid hormone, and serum ferritin | -- | Calibration plot |
|  | Zhou (2024) (22) | Cognitive test | CKD | 26.8% | Age, race, education, annual family income, body mass index, estimated glomerular filtration rate, serum albumin, and uric acid | **Int:** 0.764 (0.763, 0.807)  **Ext:** 0.752 (0.654, 0.850) | Brier Score |
|  | Ding (2025) (23) | MoCA | Hemodialysis | 76.4% | Age, sleep disorders, years of education, levels of α-Klotho, and levels of β-Klotho | **Int:** 0.894 (0.766, 0.887) | -- |
|  | Chen (2023) (18) | MoCA | Hemodialysis | 31.5% | Age (aged 55.0-64.0 years, aged 65.0-74.0 years, aged ≥75.0 years), duration of dialysis (≥5 years), and current smoker | **Int:** 0.84 (0.77, 0.91) | -- |
|  | Fan (2023) (12) | MoCA | Hemodialysis | 62.0% | Age, dialysis vintage, higher sensitive C-reactive protein, and lower albumin | **Int:** 0.892 (0.813, 0.945)  **Ext:** 0.899 (0.804, 0.958) | Calibration plot |
|  | Jiang (2023) (24) | MoCA | Hemodialysis | 65.6% | Age (>64 years old), disease duration (>4 years), education level (junior high school and below), anxiety, depression, hemoglobin (>102.74g/L), dialysis duration (>31 months), and care method (routine care) | **Int:** 0.745 (0.647, 0.811)  **Ext:** 0.895 (0.816, 0.975) | Calibration plot and H-L test |
|  | Sun (2023) (21) | MoCA | Hemodialysis | 60.0% | Hemoglobin, urea nitrogen, and mean low-frequency amplitude value in the left central posterior gyrus | -- | -- |
| CF | Cao (2025) (8) | MoCA | Hemodialysis | 14.2% | Age, mode of residence, medical payment method, exercise, alcohol consumption, dialysis vascular access, serum albumin classification, serum phosphorus classification, total cholesterol classification, blood urea nitrogen classification, malnutrition score, and depression score | **Int:** 0.911 (0.8653, 0.9494)  **Ext:** 0.832 | Calibration plot |
|  | Qin (2025) (20) | MMSE | Hemodialysis | 17.5% | Health empowerment, alexithymia, age, educational level, marital status and dialysis vintage | **Int:** 0.917 (0.881, 0.956) | Calibration plot and H-L test |
|  | Wei (2024) (26) | MMSE | Hemodialysis | 25.8% | Age, self-care ability, educational level, diabetes mellitus, hypertension, depression, sleep quality, social support, serum albumin, C-reactive protein, hemoglobin, blood creatinine, triglycerides, total cholesterol, low-density lipoprotein | **Int:** 0.945 (0.910, 0.967)  **Ext:** 0.904 (0.901, 0.938) | Calibration plot |
|  | Yi (2024) (27) | MoCA | Hemodialysis | 16.7% | The calf muscle circumference, five- times-sit-to-stand test, the sarcopenia screening scale score, and the geriatric depression scale score | **Int:** 0.842 (0.779, 0.905) | Calibration plot and H-L test |
|  | Chang (2022) (9) | MMSE | CKD | 15.2% | Advanced age, depression, low social support, Charlson comorbidity index, eGFR, and albuminuria | **Int:** 0.91 (0.89, 0.94) | Calibration plot |
|  | Luo (2022) (19) | MMSE | CKD | 21.9% | Barthel Index score, albumin, education level, 15-item geriatric depression scale score, and social support rating scale score | **Int:** 0.913 | -- |
| MCI | Xu (2024) (11) | MoCA and MMSE | Non-dialysis CKD | 50.1% | Age, educational level, occupation status, use of smartphone, sleep disorders, hemoglobin, and platelet count | **Int:** 0.928 (0.902, 0.953)  **Ext:** 0.897 (0.844, 0.950) | Calibration plot and H-L test |
|  | Yang (2024) (10) | MoCA and MMSE | Non-dialysis CKD | 50.9% | Age, educational level, occupational status, use of smartphones, sleep disorder, and hemoglobin | **Int:** 0.926 (0.925, 0.927) | Calibration plot and H-L test |
| “--”: not reported; MoCA: the Montreal cognitive assessment; MMSE: mini-mental state examination; CKD: chronic kidney disease; CI: cognitive impairment; CF: cognitive frailty; MCI: Mild cognitive impairment; Int: internal validation; Ext: external validation. | | | | | | | |

| **Supplementary Table 4. The details of model information stratified by CKD population.** | | | | | | |
| --- | --- | --- | --- | --- | --- | --- |
| CKD population | Author (year) | Cognitive assessment tools | Outcomes  （%） | Final predictors | AUC (95%CI) | Calibration |
| Hemodialysis | Cao (2025) (8) | MoCA | CF (14.2%) | Age, mode of residence, medical payment method, exercise, alcohol consumption, dialysis vascular access, serum albumin classification, serum phosphorus classification, total cholesterol classification, blood urea nitrogen classification, malnutrition score, and depression score | **Int:** 0.911 (0.8653, 0.9494)  **Ext:** 0.832 | Calibration plot |
|  | Qin (2025) (20) | MMSE | CF (17.5%) | Health empowerment, alexithymia, age, educational level, marital status and dialysis vintage | **Int:** 0.917 (0.881, 0.956) | Calibration plot and H-L test |
|  | Ding (2025) (23) | MoCA | CI (76.4%) | Age, sleep disorders, years of education, levels of α-Klotho, and levels of β-Klotho | **Int:** 0.894 (0.766, 0.887) | -- |
|  | Song (2025) (29) | MoCA | CI (77.9%) | Gender, age, protein intake, and cholesterol | -- | -- |
|  | Yi (2024) (27) | MoCA | CF (16.7%) | The calf muscle circumference, five- times-sit-to-stand test, the sarcopenia screening scale score, and the geriatric depression scale score | **Int:** 0.842 (0.779, 0.905) | Calibration plot and H-L test |
|  | Wei (2024) (26) | MMSE | CF (25.8%) | Age, self-care ability, educational level, diabetes mellitus, hypertension, depression, sleep quality, social support, serum albumin, C-reactive protein, hemoglobin, blood creatinine, triglycerides, total cholesterol, low-density lipoprotein | **Int:** 0.945 (0.910, 0.967)  **Ext:** 0.904 (0.901, 0.938) | Calibration plot |
|  | Chen (2023) (18) | MoCA | CI (31.5%) | Age (aged 55.0-64.0 years, aged 65.0-74.0 years, aged ≥75.0 years), duration of dialysis (≥5 years), and current smoker | **Int:** 0.84 (0.77, 0.91) | -- |
|  | Fan (2023) (12) | MoCA | CI (62.0%) | Age, dialysis vintage, higher sensitive C-reactive protein, and lower albumin | **Int:** 0.892 (0.813, 0.945)  **Ext:** 0.899 (0.804, 0.958) | Calibration plot |
|  | Jiang (2023) (24) | MoCA | CI (65.6%) | Age (>64 years old), disease duration (>4 years), education level (junior high school and below), anxiety, depression, hemoglobin (>102.74g/L), dialysis duration (>31 months), and care method (routine care) | **Int:** 0.745 (0.647, 0.811)  **Ext:** 0.895 (0.816, 0.975) | Calibration plot and H-L test |
|  | Sun (2023) (21) | MoCA | CI (60.0%) | Hemoglobin, urea nitrogen, and mean low-frequency amplitude value in the left central posterior gyrus | -- | -- |
| Peritoneal dialysis | Shao (2024) (25) | MoCA | CI (56.7%) | Diabetes, high levels of glycosylated hemoglobin, parathyroid hormone, and serum ferritin | -- | Calibration plot |
| Non-dialysis CKD | Xu (2024) (11) | MoCA and MMSE | MCI (50.1%) | Age, educational level, occupation status, use of smartphone, sleep disorders, hemoglobin, and platelet count | **Int:** 0.928 (0.902, 0.953)  **Ext:** 0.897 (0.844, 0.950) | Calibration plot and H-L test |
|  | Yang (2024) (10) | MoCA and MMSE | MCI (50.9%) | Age, educational level, occupational status, use of smartphones, sleep disorder, and hemoglobin | **Int:** 0.926 (0.925, 0.927) | Calibration plot and H-L test |
| CKD (unspecified stage) | Cao (2025) (28) | Health Status and Functioning Questionnaire | CI (12.8%) | Age, education level, and hemoglobin concentration | **Int:** 0.918 (0.8288, 0.977) | Calibration plot |
|  | Zhou (2024) (22) | Cognitive test | CI (26.8%) | Age, race, education, annual family income, body mass index, estimated glomerular filtration rate, serum albumin, and uric acid | **Int:** 0.764 (0.763, 0.807)  **Ext:** 0.752 (0.654, 0.850) | Brier Score |
|  | Chang (2022) (9) | MMSE | CF (15.2%) | Advanced age, depression, low social support, Charlson comorbidity index, eGFR, and albuminuria | **Int:** 0.91 (0.89, 0.94) | Calibration plot |
|  | Luo (2022) (19) | MMSE | CF (21.9%) | Barthel Index score, albumin, education level, 15-item geriatric depression scale score, and social support rating scale score | **Int:** 0.913 | -- |
| “--”: not reported; MoCA: the Montreal cognitive assessment; MMSE: mini-mental state examination; CKD: chronic kidney disease; CI: cognitive impairment; CF: cognitive frailty; MCI: Mild cognitive impairment; Int: internal validation; Ext: external validation. | | | | | | |

| **Supplementary Table 5. The details of model information stratified by Cognitive assessment tools.** | | | | | | |
| --- | --- | --- | --- | --- | --- | --- |
| Cognitive assessment tools | Author (year) | CKD population | Outcomes  （%） | Final predictors | AUC (95%CI) | Calibration |
| MoCA | Cao (2025) (8) | Hemodialysis | CF  (14.2%) | Age, mode of residence, medical payment method, exercise, alcohol consumption, dialysis vascular access, serum albumin classification, serum phosphorus classification, total cholesterol classification, blood urea nitrogen classification, malnutrition score, and depression score | **Int:** 0.911 (0.8653, 0.9494)  **Ext:** 0.832 | Calibration plot |
|  | Ding (2025) (23) | Hemodialysis | CI (76.4%) | Age, sleep disorders, years of education, levels of α-Klotho, and levels of β-Klotho | **Int:** 0.894 (0.766, 0.887) | -- |
|  | Song (2025) (29) | Hemodialysis | CI (77.9%) | Gender, age, protein intake, and cholesterol | -- | -- |
|  | Shao (2024) (25) | Peritoneal dialysis | CI (56.7%) | Diabetes, high levels of glycosylated hemoglobin, parathyroid hormone, and serum ferritin | -- | Calibration plot |
|  | Yi (2024) (27) | Hemodialysis | CF (16.7%) | The calf muscle circumference, five- times-sit-to-stand test, the sarcopenia screening scale score, and the geriatric depression scale score | **Int:** 0.842 (0.779, 0.905) | Calibration plot and H-L test |
|  | Chen (2023) (18) | Hemodialysis | CI (31.5%) | Age (aged 55.0-64.0 years, aged 65.0-74.0 years, aged ≥75.0 years), duration of dialysis (≥5 years), and current smoker | **Int:** 0.84 (0.77, 0.91) | -- |
|  | Fan (2023) (12) | Hemodialysis | CI (62.0%) | Age, dialysis vintage, higher sensitive C-reactive protein, and lower albumin | **Int:** 0.892 (0.813, 0.945)  **Ext:** 0.899 (0.804, 0.958) | Calibration plot |
|  | Jiang (2023) (24) | Hemodialysis | CI (65.6%) | Age (>64 years old), disease duration (>4 years), education level (junior high school and below), anxiety, depression, hemoglobin (>102.74g/L), dialysis duration (>31 months), and care method (routine care) | **Int:** 0.745 (0.647, 0.811)  **Ext:** 0.895 (0.816, 0.975) | Calibration plot and H-L test |
|  | Sun (2023) (21) | Hemodialysis | CI (60.0%) | Hemoglobin, urea nitrogen, and mean low-frequency amplitude value in the left central posterior gyrus | -- | -- |
| MMSE | Qin (2025) (20) | Hemodialysis | CF (17.5%) | Health empowerment, alexithymia, age, educational level, marital status and dialysis vintage | **Int:** 0.917 (0.881, 0.956) | Calibration plot and H-L test |
|  | Wei (2024) (26) | Hemodialysis | CF (25.8%) | Age, self-care ability, educational level, diabetes mellitus, hypertension, depression, sleep quality, social support, serum albumin, C-reactive protein, hemoglobin, blood creatinine, triglycerides, total cholesterol, low-density lipoprotein | **Int:** 0.945 (0.910, 0.967)  **Ext:** 0.904 (0.901, 0.938) | Calibration plot |
|  | Chang (2022) (9) | CKD | CF (15.2%) | Advanced age, depression, low social support, Charlson comorbidity index, eGFR, and albuminuria | **Int:** 0.91 (0.89, 0.94) | Calibration plot |
|  | Luo (2022) (19) | CKD | CF (21.9%) | Barthel Index score, albumin, education level, 15-item geriatric depression scale score, and social support rating scale score | **Int:** 0.913 | -- |
| MoCA and MMSE | Xu (2024) (11) | Non-dialysis CKD | MCI (50.1%) | Age, educational level, occupation status, use of smartphone, sleep disorders, hemoglobin, and platelet count | **Int:** 0.928 (0.902, 0.953)  **Ext:** 0.897 (0.844, 0.950) | Calibration plot and H-L test |
|  | Yang (2024) (10) | Non-dialysis CKD | MCI (50.9%) | Age, educational level, occupational status, use of smartphones, sleep disorder, and hemoglobin | **Int:** 0.926 (0.925, 0.927) | Calibration plot and H-L test |
| Other tools | Zhou (2024) (22) | CKD | CI (26.8%) | Age, race, education, annual family income, body mass index, estimated glomerular filtration rate, serum albumin, and uric acid | **Int:** 0.764 (0.763, 0.807)  **Ext:** 0.752 (0.654, 0.850) | Brier Score |
|  | Cao (2025) (28) | CKD | CI (12.8%) | Age, education level, and hemoglobin concentration | **Int:** 0.918 (0.8288, 0.977) | Calibration plot |
| “--”: not reported; MoCA: the Montreal cognitive assessment; MMSE: mini-mental state examination; CKD: chronic kidney disease; CI: cognitive impairment; CF: cognitive frailty; MCI: Mild cognitive impairment; Int: internal validation; Ext: external validation. | | | | | | |
